# Sniffing Out Urinary Tract Infection—Diagnosis Based on Volatile Organic Compounds and Smell Profile

**DOI:** 10.3390/bios10080083

**Published:** 2020-07-23

**Authors:** Valentin-Mihai Dospinescu, Akira Tiele, James A. Covington

**Affiliations:** 1 Warwick Medical School, University of Warwick, Coventry CV4 7AL, UK; V.Dospinescu@warwick.ac.uk; 2 School of Engineering, University of Warwick, Coventry CV4 7AL, UK; F-A.Tiele@warwick.ac.uk

**Keywords:** urinary tract infection (UTI), electronic nose (eNose), volatile organic compound (VOC), ion mobility spectrometry (IMS), gas chromatography–mass spectrometry (GC-MS), pattern recognition, metabolite detection

## Abstract

Current available methods for the clinical diagnosis of urinary tract infection (UTI) rely on a urine dipstick test or culturing of pathogens. The dipstick test is rapid (available in 1–2 min), but has a low positive predictive value, while culturing is time-consuming and delays diagnosis (24–72 h between sample collection and pathogen identification). Due to this delay, broad-spectrum antibiotics are often prescribed immediately. The over-prescription of antibiotics should be limited, in order to prevent the development of antimicrobial resistance. As a result, there is a growing need for alternative diagnostic tools. This paper reviews applications of chemical-analysis instruments, such as gas chromatography–mass spectrometry (GC-MS), selected ion flow tube mass spectrometry (SIFT-MS), ion mobility spectrometry (IMS), field asymmetric ion mobility spectrometry (FAIMS) and electronic noses (eNoses) used for the diagnosis of UTI. These methods analyse volatile organic compounds (VOCs) that emanate from the headspace of collected urine samples to identify the bacterial pathogen and even determine the causative agent’s resistance to different antibiotics. There is great potential for these technologies to gain wide-spread and routine use in clinical settings, since the analysis can be automated, and test results can be available within minutes after sample collection. This could significantly reduce the necessity to prescribe broad-spectrum antibiotics and allow the faster and more effective use of narrow-spectrum antibiotics.

## 1. Introduction

A urinary tract infection (UTI) is a microbial condition that can affect any part of the urinary system. UTI is one of the most common bacterial infections with 150 million cases per year worldwide [1]. Adult women are 30 times more likely than men to develop a UTI [2]. Moreover, it is estimated that 50–60% of women will experience at least one episode of UTI during their lifetime [1] with the prevalence increasing with age (20% in women over 65 and overall population prevalence of 11%) [3,4]. UTI is a major global health burden, causing great distress as it is very uncomfortable and impedes the patient’s ability to conduct day-to-day activities, such as work [3,5,6].

Current diagnostic methods rely heavily on a urine dipstick test, which offers a fast result (available in 1–2 min) but has a low positive predictive value [7]. The accepted alternative method involves culturing of the pathogens, which is more accurate and can inform clinicians on the type of bacteria present. This is time-consuming and delays diagnosis (24–72 h between sample collection and pathogen identification) [4]. Broad-spectrum antibiotics are therefore often prescribed immediately. However, due to the delay, this practice is fuelling antimicrobial resistance. As a result, there is a growing need for alternative diagnostic tools. Recent advances in chemical-analysis technologies suggest that it might be possible to ‘sniff out’ medical conditions, including bacterial infections, by analysing volatile organic compounds (VOCs) emanating from the body and its waste.

The use of smell is possibly one of the earliest forms of medical diagnosis. Both ancient Chinese and Greek physicians already recognised its diagnostic potential [8]. The father of modern medicine, Hippocrates, used to put sputum samples on hot coals to generate an odour, then, based on the smell, apply different treatments [9,10]. The basic concept of using odours to distinguish between disease states has been supported by studies utilising the olfactory abilities of well-trained canines [10,11]. Numerous studies have demonstrated the ability of canines to smell cancers in the bladder [12], lung [13], breast [14], prostate [15], and ovaries [16]. Despite their abilities, medical detection dogs are very expensive to train and are impractical for routine clinical use.

It is now understood that odours mainly comprise of volatile organic compounds (VOCs), which are carbon-based compounds, with high vapour pressure and low boiling points. VOCs are produced within the body as a result of metabolic processes from the host, from microbial pathogens, or resulting from a host response to pathological processes, such as infection or inflammation [17]. VOCs in urine are considered intermediate or end products of metabolic pathways [18]. These occur in relatively higher concentrations than in other medium, such as breath—which makes them easier to detect [19]. Recent studies have shown that characteristic urine VOC profiles can be linked to infectious diseases [20,21] and different types of cancers [22,23,24]. These studies have demonstrated the potential in utilising urine for diagnostic purposes. 

The objective of this article is to review previous studies, focusing on the utility of urine headspace VOC analysis as a diagnostic tool for UTI. This review first discusses UTIs and critically evaluates the current diagnostic approach. Next, the paper reviews the analytical platforms that have been evaluated as potential diagnostic tools for UTIs and summarises their characteristics. An extensive list of VOCs associated with common UTI pathogens, either in cultures or urine samples, is then presented. Finally, the developments needed to bring this approach into clinical practice are discussed. 

## 2. Urinary Tract Infection

UTI can affect any part of the urinary system—kidneys (pyelonephritis), uterus, bladder (cystitis) and urethra (urethritis). Most UTIs involve the lower urinary tract (bladder and urethra). The infection can be caused by bacteria (Gram-negative and Gram-positive), as well as fungi. Furthermore, 70% of community-acquired UTIs and 50% of hospital-associated UTI are caused by UroPathogenic *Escherichia coli* (UPEC) [25,26]. 

UTIs can be classified as complicated or uncomplicated. When cystitis or pyelonephritis occurs in an otherwise healthy individual (premenopausal and non-pregnant women) and no abnormalities are present in the urinary tract, the UTI is considered uncomplicated [27]. Immunocompromised individuals, pregnant women, or patients with urinary tract abnormalities and UTI are classified as complicated [28]. Abnormalities associated with complicated UTI include obstructions (tumours of the urinary tract), instrumentation (indwelling urethral catheter) or metabolic abnormalities (renal failure) [28]. If a UTI is not treated, the infection could spread from the kidneys into the bloodstream, causing bacteraemia. If a systemic inflammatory response occurs, this could potentially be followed by septicaemia, which can be fatal [1,29,30].

Even though UTI is rarely fatal, it produces high morbidity in vast numbers of patients. A patient with cystitis often presents with urinary symptoms, which include dysuria (painful urination), urinary frequency, urgency or haematuria (blood in urine) [1]. As the onset of UTI is sudden, rapid and painful, it often incapacitates the person from their normal day-to-day activities, creating a feeling of guilt and even depression [6]. Both physical and social functioning are negatively affected [3]. The direct impact of UTI on health care relates to the cost of prescriptions, hospital expenses, travel, medical visits and diagnosis—sometimes multiple urine cultures and imaging studies are required [31]. Overall, it results in lowering the patient’s quality of life. 

Most commonly, UTI is initiated when a urinary pathogen from the bowel (sometimes from the vagina—inoculation during sexual activity) enters the urinary tract via the urinary meatus [27]. In uncomplicated UTI, following UPEC, the most common pathogens are: *Klebsiella pneumoniae*, *Staphylococcus saprophyticus*, *Enterococcus faecalis*, group B *Streptococcus*, *Proteus mirabilis*, *Pseudomonas aeruginosa*, *Staphylococcus aureus*, and *Candida* spp [1]. In complicated UTI, the most prevalent agents of infection after UPEC are: *Enterococcus* spp., *K. pneumoniae*, *Candida* spp., *Staphylococcus aureus*, *P. mirabilis*, *P. aeruginosa*, and group B *Streptococcus* [1].

Broad-spectrum antibiotics are generally used for treating UTIs. However, with a better understanding of the adverse impact this has on commensal bacteria present in the gut microbiome, this practice is becoming of concern to healthcare professionals [32]. Moreover, the over-prescription of broad-spectrum antibiotics could accelerate the development of antimicrobial resistance (AMR). It is estimated that, by 2050, AMR will cause 10 million deaths globally (more than cancer—8.2 million) [33]. The threat to patients is acquiring a multidrug-resistant agent, i.e., a microorganism that shows antimicrobial resistance to at least one antimicrobial drug in three or more antimicrobial categories. In recent years, the number of multidrug-resistant Gram-negative bacteria have increased and have been found to cause UTIs [34]. Furthermore, antibiotics increase inflammation in the gut, which promotes the proliferation of *E. coli* [35]. Since UPEC is usually found in the gastrointestinal tract, the surge in proliferation increases the risk of subsequent UTI recurrence. As a result, broad-spectrum antibiotics can be used to treat UTIs, but this also increases the risk of developing UTIs in future. UK NICE guidelines (National Institute for Health and Care Excellence) recommends the use of narrow-spectrum agents for UTIs whenever possible. This approach would be feasible if the pathogen could be rapidly identified.

## 3. Current Diagnostic Methods for Urinary Tract Infection

The diagnosis of UTI is currently based on culturing samples in a centralised clinical laboratory, which results in a delay of 24–72 h from the moment the patient provides the sample to identifying the bacteria. UTIs are usually defined by the urinary symptoms and the presence of at least 10^4^ CFU/mL in urine, though the cut-off point differs (NHS considers 10^5^ CFU/mL as positive and the threshold changes based on the presented symptoms) [25,36]. 

In the 1980s, the urine dipstick test was introduced to provide a fast, cheap and useful test in diagnosing UTI. The dipstick test is a qualitative test, which assesses the presence of leukocyte esterase and nitrate. Leukocyte esterase is a protein made by white blood cells and acts as a biomarker for those immune cells. The presence of leukocytes in urine is indicative of a UTI. Nitrite is found in urine due to the presence of nitrate reducing bacteria. Even though the dipstick is a cheap and fast test, it does not narrow down the infection to a causative agent and may even miss some infections (e.g., non-nitrate reducing bacteria). In a meta-analysis, the overall sensitivity of the urine dipstick test for leukocyte esterase was 48–86%, with a specificity of 17–93%. For the nitrate dipstick test, the sensitivity was even lower, with values between 45% and 60%, and a specificity of 85–98%. Overall, when combining both tests, the sensitivity increases to 68–88% and specificity to 62–97% (one or both positive), depending on the brand, cut-off standard, and population demographic [37]. A similar study found a sensitivity of 75% and specificity of 82% [38].

A rapid point-of-care test, which can accurately identify the pathogen, would have a positive impact on UTI treatment, since the most effective course of antibiotics could be initiated immediately, instead of prescribing broad-spectrum antibiotics. This would provide a significant benefit to the patient in terms of saved time and avoided unnecessary pain, since they do not need to wait on culture results. Furthermore, a standardised sensor would remove human error, making diagnosis faster, easier, and objective. 

## 4. Volatile Organic Compounds Detection Technology and Data Analysis

**Figure 1:**
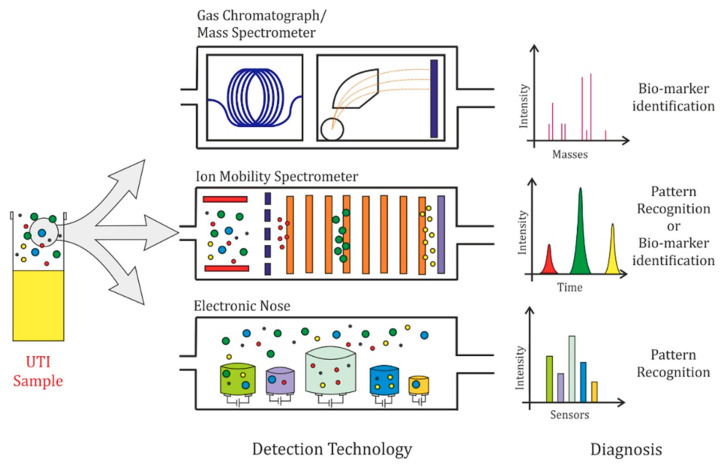
Volatile organic compound (VOC) detection technologies for analysis of urinary tract infection (UTI) urine samples; (**Top**) gas chromatography—mass spectrometry (GC-MS): the column of the GC is represented in blue, resulting in compound separation based on their physical properties and mass; (**Middle**) ion mobility spectrometry (IMS): the separation of compounds based on ion mobility is represented by different groups of VOCs clustering together; (**Bottom**) electronic nose (eNose): the eNose response is based on an array of different cross-sensitive gas sensors. A typical response is shown for each method. VOCs are illustrated as green, blue, yellow and red circles.

There are various competing technologies available for the analysis of VOCs in urine. The most commonly used technique is gas chromatography, coupled with mass spectrometry (GC-MS) [18]. Other methods include proton transfer reaction mass spectrometry (PTR-MS), ion mobility spectrometry (IMS), selected ion flow tube mass spectrometry (SIFT-MS), field asymmetric ion mobility spectrometry (FAIMS), gas chromatography flame ionisation detection (GC-FID), and electronic noses (eNoses), which are made up of arrays of sensors, e.g., metal oxide (MOS), piezoelectric, or conducting polymer (CP). A summary of the most common technologies, with a visual explanation of their working principles, is shown in Figure 1. 

In GC-MS, the GC part involves injecting samples into a separation column, where they are transported by an inert carrier gas (usually helium or hydrogen). The gaseous mixture is separated into its components based on the relative interactions between the analytes and the inner coating of the column. Molecules with column interactions (e.g., lower boiling points or weight) exit the column before higher interaction compounds. The MS is then used to detect exiting compounds. An ion source is used to produce analyte ions. Electron impact ionisation is a commonly used technique, which involves bombarding the analytes with a beam of electrons. This is followed by a mass analyser, which sorts the fragment ions by their mass-to-charge ratio [39]. Lastly, the ions reach a detector (e.g., electron multiplier), which measures the ionised mass fragments as an electrical signal. GC-based technologies may also include two-dimensional GC-MS (GCxGC-MS) or combining GC with a time-of-flight mass spectrometer (GC-TOF-MS). In a GC-TOF-MS system, once the sample has passed through the GC column, the separated molecules are ionised and enter the TOF ‘flight box’. TOF-MS works on the principle that when molecules are exposed to a pre-determined amount of energy, larger, heavier ions will take longer than smaller, lighter ions to travel a set distance [40].

An emerging alternative to GC-MS is IMS. This technique uses a high electric field at ambient pressure to separate different ions formed from the target analyte, based on differences in their mobility. Drift-tube IMS uses the time taken for ionized molecules to traverse the tube. These ions are driven along the tube by a constant high-voltage electric field. Against the flow of ions, a buffer gas (i.e., nitrogen) is used slow down the ions through collisions. This produces a time-dependent signal that corresponds with ion mobility, with its drift time being a function of the number of collisions and the ion interaction with the electric field. The drift-tube IMS method can be used in combination with GC as a pre-separator. FAIMS is a different form of IMS, which reduces the drift tube length by applying an alternating electric field across the ion path, changing the differential mobility of each ion. FAIMS has been used to diagnose various diseases including colorectal cancer [41,42]. IMS systems were originally used to detect chemical warfare agents, explosives, and illegal drugs, but have found increasing applications in the medical domain. [43]. 

An eNose is an instrument that attempts to replicate the function of the biological olfactory system by measuring VOCs as a whole, rather than identifying specific chemicals. It uses an array of broadly tuned (cross-sensitive) gas sensors, which are used to measure a wide range of VOCs. As each sensor in the array is different, the response between the odour and the sensor will be unique within the array. This creates an odour “fingerprint” that can be learnt by some form of pattern recognition technique. The eNose instrument types most frequently used for diagnostic applications in detecting human disease include sensor arrays made up of metal oxide sensors (MOS), electrochemical sensors (EC), conducting polymers (CP), carbon black polymer composite (CBPC), gold nanoparticles (GNP), quartz crystal microbalance (QMB) sensors, and optical sensors [44]. 

MOS sensors are based on a reduction-oxidation reaction. In ‘clean’ air, oxygen is absorbed onto the gas-sensitive layer of the sensor (e.g., tin oxide, SnO_2_). When exposed to a reducing or oxidising gas, the oxygen reacts to the target gas that changes the conductivity of the sensitive layer [45]. EC sensors are based on an electrochemical oxidation or reduction reaction, which occurs at the working electrode (WE). In the presence of VOCs, the catalytic material reacts, and produces a current at WE. A counter electrode (CE) keeps the sensor electrically neutral [46]. The operation of conducting polymer (CP) gas sensors is based on a change in conductivity of the polymer film, when exposed to analyte vapours [47]. CBPC sensors operate based on swelling of the polymer upon exposure to target gases, which in turn changes the resistance of the conducting film [48]. GNP sensors comprise of monolayer-capped gold nanoparticle chemiresistive films [49]. When exposed to VOCs, they diffuse into the sensing film and react with the capped ligands/functional groups, causing a volume shrinkage/expansion in the nanomaterial film, resulting in a change in resistance [49,50]. QMB sensors use highly sensitive quartz crystals for the measurement of the mass and viscosity of VOCs [51]. Changes in gravitational load on the sensor and viscoelastic properties of the VOCs cause a frequency shift of the quartz crystal resonator [52]. The optical sensor principle is based on absorption spectroscopy, using infrared (IR). The amount of absorption depends on the gas concentration, at a wavelength related to a target gas—selected using optical filters [53]. 

Each of these sensor technologies are associated with advantages and disadvantages. MOS sensors are low cost and highly sensitive but have a limited sensing range and typically operate at higher temperatures (between 300–500 °C) [54]. This high temperature requirement can result in increased power consumption, while EC, CP, CBPC, GNP, and QMB sensors operate at ambient temperature and therefore require lower power. CP and CBPC sensors are inexpensive and are highly sensitive to many VOCs, but have a shorter operational life, compared to MOS sensors. They also demonstrate poor repeatability and reproducibility due to the random nature of the polymer [55]. EC sensors have fast response times, but are bulky in size and have limited sensitivity to simple or low molecular weight gases [56]. GNP sensors are newer and combine gold nanoparticles with polymers or metal-oxides. The polymer variants operate at room temperature but suffer from similar issues to CBPC sensors. The metal-oxide composite variants operate at elevated temperature and have better selectivity than normal MOS sensors, but are more challenging to fabricate [57,58]. QMB sensors have good precision for a diverse range of VOCs, but require complex circuitry, have a poor signal-to-noise ratio, and are sensitive to humidity and temperature [56]. Optical sensors have very fast response times, high sensitivity and selectivity (especially to gases such as CO_2_ and CH_4_), but are more expensive, difficult to miniaturise and may require complex electrical circuits [56]. In order to take full advantage of these characteristics, a combination of multiple sensor technologies can be deployed in a single device.

Recently, at the University of Warwick, the WOLF (Warwick olfaction) eNose was developed, which contains 13 multi-technology sensors [59]. It was successfully used to distinguish between colorectal cancer (CRC) and irritable bowel syndrome (IBS) patients [59]. While eNoses have been emerging over the past decades as a new way to diagnose a variety of complex illnesses [60], they also have downsides. Due to the nature of the sensors, they often drift over time and the response is dependent on humidity and temperature [61]. As the sensors age, chemical deterioration can damage the detecting surface of the sensor, which can cause issues with reproducibility. Without proper maintenance, even if an identical sample is tested twice, the readings could be different. Approaches to mitigate against these problems usually involve repeated sensor calibration [62]. 

In SIFT-MS, analytes are introduced into a flow tube and ionised with chosen precursor ions (usually H_3_O^+^, NO^+^ or O_2_^+^). The resulting ions are quantified by mass filtering, using a quadrupole MS to separate the ions based on their mass-to-charge ratio. SIFT-MS has the benefit of detecting compounds and calculating the concentration from the ratio of product to precursor ions present in the headspace of the sample in real-time, as well as not requiring calibration.

Other methods, such as nuclear magnetic resonance (NMR) spectroscopy and proton transfer reaction-mass spectrometry (PTR-MS) have been used as chemical-detection technologies for VOC identification in research, but also for diagnosis [60,63,64,65,66]. In NMR spectroscopy, the atomic nuclei are excited by radio waves, resulting in nuclei orientation changes along the magnetic field. When excitation is stopped, the return to the initial lower energy orientation causes energy emissions that can be detected. This provides insight into the chemical composition of the sample [67]. In PTR-MS, VOCs undergo proton-transfer reactions (with H_3_O^+^ ions) in a drift tube reactor and are measured by a quadrupole MS (similar to SIFT-MS) [64]. PTR-MS offers real-time analysis and has been used to test breath and urine samples for diagnosing diseases such as lung cancer and *H. pylori* infection [63,64,68]. 

Overall, eNoses and IMS have a lower purchase price, running and maintenance cost compared to GC-MS, SIFT-MS and PTR-MS. In addition, eNoses and IMS systems can be made portable and used in point-of-care applications, unlike other methods, which require laboratory settings. The disadvantages of eNoses and, in most cases, IMS are that they cannot provide an insight into the chemistry of the studied sample (e.g. chemical identification). Using GC-MS, more information about the organism growth phase and metabolic pathway can be ascertained. However, for the diagnosis of UTI, eNoses and IMS systems are still the most suitable candidates, since they are easy to use, portable, relatively low-cost, and have methods which can be automated. Table 1 provides an overview and comparison of the characteristics associated with commonly used VOC detection technologies. 

Most applications of VOC analysis aim to discriminate between disease states. This may include a simple case-control design (e.g., disease vs. controls) or a variety of investigated states and groups. These applications predominantly rely on classification analysis to distinguish between groups and to build and train models which can be used as a diagnostic tool. The classification methods most commonly used in VOC analysis include principal component analysis (PCA), linear discriminant analysis (LDA), partial least-square discriminant analysis (PLS-DA), and machine learning (ML) algorithms. PCA is an unsupervised linear method, which reduces the dimensionality (number of features, e.g., eNose sensor responses or GC-MS peaks) of the data by selecting a small number of linearly uncorrelated principal components that explain the majority of the variation in the data [69]. LDA is a supervised linear method that tries to find a linear discriminant function to separate between study groups [70]. PLS-DA is similar to LDA but attempts to maximise the co-variance between a dummy class vector and the data matrix. PCA, LDA, and PLS-DA have been applied frequently for VOC analysis studies, for both eNose and GC-MS data [71]. The disadvantage of these methods is that they are most effective for investigating linear relationships between samples and classes (e.g., disease status). However, they not well-equipped for representing non-linear relations that are likely to be present in biological samples [72]. Non-linear statistical learning approaches, based on ML, are therefore being increasingly used for the analysis of urine VOC datasets [24,73,74,75,76]. These methods can be broadly categorised into three groups: tree-based methods (e.g., random forest—RF), neural networks, and kernel-based methods (support vector machines—SVMs). The advantage of linear techniques is that they are simple to interpret, require few parameters to be optimised, and are not computationally demanding. The converse is true of ML techniques [70]. 

## 5. Detection of Urinary Tract Infection Using Volatile Organic Compounds

For urine analysis, the urine sample is usually transferred to a vial/container and then sealed. The unoccupied area/air above the urine sample is known as the headspace and VOCs from the urine desorb out of the liquid phase, into this air space and can be collected for analysis. In 1976, the prospect of using headspace analysis for identifying bacteria became apparent when Hayward et al. determined that *Proteus* species produce methyl mercaptan and dimethyl disulfide from methionine [77]. Furthermore, *P. vulgaris*, *P. mirabilis*, *P. moraganii*, and *P. rettgeri* were shown to produce benzaldehyde. In the culture filtrate, phenylacetaldehyde and 2-phenylethanol are produced from phenylalanine. Isobutyraldehyde and isobutanol are produced from valine and isovaleraldehyde, together with isoamyl alcohol, from leucine. In uncomplemented media, isobutyraldehyde, isovaleraldehyde, and isoamyl alcohol were detected. Moreover, it was shown that *E. coli* ferments lactose or arabinose for energy and produces ethanol as a by-product. 

In a paper from 1977, Hayward et al. tried to identify *E. coli* and *Proteus* species from cultures and simulated urine infections with isolated bacteria, using gas-liquid chromatography. Ethanol was suggested as an indicator for *E. coli* and dimethyl disulfide, together with methyl mercaptan, for *Proteus* species [78]. Further work by Hayward and Jeavons involved testing 75 clinical urine samples. It was found that ethanol is a biomarker for *E. coli* when grown in the presence of lactose and dimethyl disulfide (as well as methyl mercaptan) for *Proteus mirabilis* and *Proteus inconstans* A [79]. The analysis can be done in 3–5 h, after urine incubation. One drawback is the inability to tell apart *Klebsiella aerogenes* from *E. coli*, since both species ferment lactose to ethanol. In a similar study, 49 strains of *E. coli* were grown in media supplemented with arabinose [80]. It was confirmed that the bacteria produce ethanol with a maximum concentration achieved after eight hours. As expected, the quantity of ethanol production is directly proportional to the total number of bacteria present in the culture, thereby providing a way to estimate the bacterial load in clinical samples. Following this, the possibility of diagnosing UTIs instigated by another organism was investigated using a similar method. *P. mirabilis* was found to produce dimethyl disulfide from methionine and it was once again confirmed that *E. coli* produces ethanol from arabinose in culture [81]. The advantage of growing these bacteria in enriched media, with arabinose and methionine, is that diagnosis is possible in just four hours. However, in the urine cultures containing *K. aerogenes* or *K. ozaenae,* ethanol was found in similar quantities as those produced by *E. coli* showing the decreased discriminatory power of this method [79,81]. 

Discrimination between *E. coli* and *Klebsiella* species using the production of ethanol from lactose (*E. coli*) or adonitol (*Klebsiella*) is also possible in less than six hours [82]. In addition, the following VOCs: trimethylamine, ethanol and ethyl acetate, were associated with *P. mirabilis, P. vulgaris*, *P. rettgeri*, and *P. inconstans* found via GC-MS [83]. During these experiments, it was confirmed that *K. aerogenes* and *E. coli* produces ethanol as well as ethyl acetate. It was also shown that ethyl acetate was produced by *S. faecalis*, N-propanol in *E. coli*’s headspace, and that ethyl acetate was found in *P. inconstans* B [83]. 

These developments have paved the way to show that VOC analysis of headspace can be used to detect UTIs. This technique has three benefits: (1) it can provide chemical insight of the organism tested, (2) it can indicate the metabolic pathway used by the bacteria, and (3) indicate its growth phase. Unfortunately, this method has not gained wide-spread use in clinical settings. One reason for this could be the complexity of analysing VOCs found in the headspace of bacterial cultures. These VOCs often overlap between species, which makes discrimination very challenging. 

Storer et al. used SIFT-MS to monitor 21 compounds, of eight different organisms: *E. coli*, *P. aeruginosa*, *S. aureus*, *P. vugaris*, *K. pneumoniae*, *E. faecalis*, *S. epidermidis*, and *C. albicans*. It was shown that this technology could identify a UTI in urine samples [84]. However, the urine was incubated with the bacteria for six hours, allowing for compounds to build-up in the headspace. Furthermore, the number of compounds measured did not facilitate discrimination between the different species. In contrast, a paper by Thorn et al. showed that *E. coli*, *S. aureus*, *P. mirabilis*, *Burkholderia cepacian,* and *Streptococcus pyogenes* could be distinguished by SIFT-MS. They monitored a set of 25 VOCs, out of which 15 were the same as the aforementioned paper [85]. This highlights both the advantage and disadvantage of this technology, i.e., the bacteria can indeed be identified, if sufficient data are available. At the same time, prior knowledge of the combination of VOCs is required to discriminate between the species. Furthermore, SIFT-MS has a high entry price point, costing even more than GC-MS. 

Recently, GCxGC-TOF-MS methods were used to discover 28 new *P. aeruginosa* VOCs as well as confirming 28 former VOCs [86]. This area of research seemed to gain further interest between 2017–2019, since a number of works relating to this topic were published [87,88,89,90,91,92,93]. Most research groups used a metabolite detection approach: GC-MS [87,88], GCxGC-TOF-MS [93], and TD-GC-MS (GC-MS coupled with a thermal desorption, TD, system) [91]. DeJong et al. identified *E. coli* in human urine (simulated infection samples) using surface enhanced Raman spectroscopy [92]. 

As demonstrated, metabolite detection can be used to determine the pathogen present in urine samples. Another application is determining its antibiotic resistance. Ion-molecule reaction-mass spectrometry (IMR-MS) has been utilised to investigate whether *E. coli* was ampicillin resistant and *S. aureus* oxacillin resistant [94]. This was achieved by using the concentration of methanethiol as a proxy of growth in culture, in the presence or absence of antibiotics. 

The various VOCs discovered to be produced by the most common UTI pathogens (in either cultures or urine samples) are shown in Table 2. There are overlaps between the species, such as ethanol, ethyl acetate, and methyl mercaptan, which are by-products of the bacterium metabolism.

The same pathogens responsible for UTI can also cause infections in other parts of the body, such as the lungs, blood, or burn wounds. The fact that VOCs identified in urine specimen culture, or pure culture, overlap with VOCs found in the headspace of the same bacteria from different clinical specimens confirms that the bacteria is being detected. For example, 2-aminoacetophenone is the compound responsible for the grape-like odour of *P. aeruginosa* and was found from clinical isolates from the Burn Unit of the University of Iowa Hospitals. Furthermore, 2,4-Dimethylquinazoline and 4-Mehtylquinazoline were also found [111]. In addition, SIFT-MS was used on cultures of *P. aeruginosa* from cystic fibrosis patients [112]. Simulated blood infections and blood culture bottles with *E. coli*, *P. aeruginosa* and *S. aureus* were analysed by SIFT-MS, confirming previously found products [113,114,115], with antibiotic resistance of *E. coli* to gentamicin and *S. aureus* to flucloxacillin, determined by VOC suppression in culture [113]. *E. coli* resistance to gentamicin was determined using SIFT-MS—after 22 h of incubation, the production of hydrogen sulfide from the bacteria was eliminated (acetic acid, 2-aminoacetophenone, ethanol, methanethiol, pentanols, propanol, and dimethyl disulfide were also inhibited). When *S. aureus*’s headspace was analysed, 2-aminoacetophenone, ethanol and formaldehyde production were found to be inhibited by flucloxacillin [113]. GC-MS has been used on infected sinus mucus with bacterial species including *S. aureus* and *P. aeruginosa* [103]. In the lung of cystic fibrosis patients, colonized with *P. aeruginosa*, 2-aminoacetophenone was found by GC-MS breath analysis, but also in the headspace of the cultured organisms [116]. The headspace of *E. coli*, *P. aeruginosa*, *K. pneumoniae*, and *S. aureus* from inoculated artificial sputum medium or nutrient broth was tested with GC-TOF-MS [117]. Similarly, GC-IMS has been deployed to analyse the headspace of blood cultures containing *E. coli*, *S. aureus* and *P. aeruginosa* [118]. A study involving 210 samples of bronchoalveolar lavages found volatile fatty acids in the headspace of *Candida alibicans*, *Enterococcus faecalis*, *Pseudomonas aeruginosa*, *K. pneumoniae*, *Proteus* species, *S. aureus,* and other bacteria [119]. Using clinical blood cultures and reference strain bacterial blood cultures, volatile fatty acids were detected in a number of UTI relevant pathogens [120]. IMR-MS was also used to distinguish between Gram-negative and Gram-positive bacteria from positive blood culture samples, with some of the bacteria also being responsible for UTI: *S epidermidis* (*n* = 87), *S. aureus* (*n* = 17), *E. coli* (*n* = 30), and *K. pneumoniae* (*n* = 8) [121]. 

## 6. Electronic Nose and Ion Mobility Spectrometry Use

There have been a number of studies that utilised eNoses to detect UTI. In 2001, the Osmetech Microbial Analyzer (OMA) polymer sensor array was used directly on 534 urine samples and discriminated between infected from non-infected samples, at a cut-off of 10^5^ CFU/mL, with a sensitivity of 83.48% and specificity of 87.59% using principal component analysis (PCA) [122]. 

Despite these promising results, the drawback of this study is that it only provides information about the infection status of the patient, without identifying the causative agent. The following year (2002), a different eNose system (the Bloodhound BH114) was used to not only identify UTIs but also the bacterial agent [123]. A limitation of this study was that samples were cultured for 4.5 or five hours, making the sample processing time similar to the previously discussed GC-based work, without providing chemical identification. A total of 45 samples were analysed, including 30 UTI cases. Using genetic algorithms and back-propagation neural networks, discrimination between *E. coli*, *Proteus* spp., and coagulase negative *Staphylococcus* spp. was possible with 98% prediction rate (31 training samples and 14 tests with 13 out of 14 correctly identified) or 95% prediction rate (26 training samples and 19 tests with 18 out of 19 recognised) depending on which neural network was applied. Another study in 2008 by Kodogiannis et al. demonstrated that the Bloodhound BH114 can be used to discriminate the same species, this time, achieving 100% accuracy with four feature extraction approaches (divergence, absorption, desorption, and area) [124]. 

Similarly, the Cyranose Sciences’ Cyranose 320 eNose was used in parallel with an Agilent 4440 Chemosensor mass spectrometer to discriminate between infected and healthy urine samples, without any prior culturing [125]. One of the problems faced when using this setup is the amount of data generated in a single experiment. As such, the paper investigated different data reduction approaches including multilayer perceptron, radial basis function (RBF) neural networks and autoregressive exogeneous models. In the urine screening experiments, 189 sensor responses from the Cyranose 320 eNose were used, with an ARX (autoregressive exogenous type) model, to achieve an accuracy of 65% with data from all 32 sensors and 67% using 19 sensors (most negatively correlating). Furthermore, 71% accuracy was achieved when that was coupled with normalising. The accuracy obtained using another approach (RBF) was 50%, with correlation results of 65% and 80% with the Hybrid NARX model (73% was achieved when sensor numbers were reduced) [125]. 

Further studies have been conducted since 2011 [84,85]. Sabeel et al. used the Cyranose 320 eNose to analyse urine containing either bacteria (one sample) or mucus (six samples), compared to healthy urine (two samples). The drawback of this study is the small sample size and that no data accuracy was provided [126]. In a later study, the ChemPro 100i (a device combining a type of IMS with MOS gas sensors) was used in a proof-of-principle study with 101 bacterial cultures from urine containing: *E. coli*, *S. saprophyticus*, *Klebsiella* species as well as *E. faecalis* [127]. The ChemPro100i achieved a sensitivity and specificity of 90% and 96%, respectively, (using linear discriminant analysis—LDA) to identify the bacteria and 95% and 97%, respectively, to distinguish between healthy and infected samples (using logistic regression) [127]. This study demonstrated that accurate discrimination between healthy and infected urine is possible, when different bacteria is present, using pattern recognition techniques. The main advantage of using ChemPro 100i for UTI diagnosis is that the device is portable (handheld), thereby facilitating point-of-care use. 

In a study by Ratiu et al., the ChemPro 100i was used to discriminate between three bacterial species (*E. coli*, *Bacillus subtilis* and *S. aureus*) [91]. In more recent work by Adebiyi et al., a new eNose, produced by IBM, called the Electronic Volatile Analyzer (EVA) was used to differentiate strains of *E. coli*-inoculated urine. A key feature of the new eNose is that it is intended for internet-of-things (IoT) applications. By using different statistical methods, various accuracies were achieved by the EVA eNose with, on average, over 90%, and some with even 100% [90]. Overall, current developments in pattern recognition devices suggest that it is possible to detect specific bacteria in the VOC headspace of urine. Table 3 provides a summary of these developments.

## 7. Discussion and Conclusions

UTI diagnosis has not changed for over 10 years and is not reflective of the currently available methods and technologies. There is a clear need for a more accurate and rapid test, which is economically feasible. Several research teams across the world are working on better ways to diagnose UTI in the pursuit of the £8 m Longitude prize [131], an inducement prize contest by the UK-based charity NESTA (National Endowment for Science, Technology and the Arts), incentivising the development of an affordable, accurate, and fast point-of-care test for bacterial infection. This competition highlights both the clinical demand for a new diagnostic tool and the growing research interest in the field. 

There are several challenges associated with UTI diagnosis based on VOC analysis. While GC-MS is considered the gold standard [132], this method is expensive, time-consuming and requires highly-trained operators [133]. This also applies to other technologies, such as PTR-MS and SIFT-MS. Most analytical platforms are not portable and therefore unsuitable for point-of-care applications. As a result, urine samples need to be stored and transported from a clinical to a laboratory setting. Thereafter, the urine sample often still has to be cultured (3.5–6 h). Further delays might be incurred between generating results and reporting these to the clinician. Even once the sample has been analysed and results are available, they are unlikely to be definitive, since VOCs are not always specific to a single species. Some of these delays could be reduced by utilising eNose and/or IMS technologies, which are suitable for direct analysis and can be used in a clinical setting. Other challenges associated with urine VOC analysis are more fundamental. While urine analysis is the third major diagnostic screening test in clinical laboratories, there is a lack of well-established and standardised methods regarding procedures for collection, transportation, sample preparation and analysis [134]. During sample collection, patients suspected for UTI are instructed to deliver a mid-stream urine (MSU) sample, by voiding firstly into the toilet and secondly into a urine container. MSU samples are associated with significantly higher bacterial levels than first-voided urine samples [135]. However, instructing a patient in MSU collection can be time-consuming, challenging, and can introduce bias due to incorrectly collected samples [136]. In addition to this, urine is susceptible to other confounding factors, such as dietary intake, diuresis and exercise [137], as well as intake of prescription and non-prescription medication and supplements [138]. Further difficulties are experienced during sample storage and transport. Once the sample has been provided, it is essential for the sample to be frozen immediately, or at least chilled. If the sample is left at room temperature beyond four hours before analysis, it may lead to increased numbers of colony forming units (CFU) being interpreted as significant growth [136]. The samples also need to remain frozen during transport, which can impose logistical challenges for both in-house and external sample transportation. A common solution to this is using dry ice, since it has little effect on the samples for short transport periods [139]. The age of the sample should also be considered [140]. Improper storage can lower the quality/performance of the urinary test [137]. In regard to sample preparation, various methods have been used to defrost samples. For example, frozen samples might be stored at –80 °C and thawed at room temperature [141] or stored at –20 °C and defrosted by immersing the vial in a water bath at 60 °C for 30 s [24]. Once the samples have been defrosted, an aliquot of the sample is transferred into a vial. The analysed urine volume, per sample, varies significantly across urine VOC studies, e.g., 0.75–8 mL [24,42,134,142]. If headspace analysis is to be performed, the temperature of the sample is commonly elevated, to increase the sample vapour pressure [143]. The duration and temperatures for this procedure vary across studies, e.g., 5–20 min and 37–90 °C [42,144,145,146]. Lastly, while urine constitutes a highly complex and rich source of analytes, headspace gas samples are unlikely to represent the full metabolite content of the medium. Method development studies are therefore necessary to determine what procedures should be applied in order to demonstrate the full potential of urine analysis for UTI diagnosis. Some challenges associated with sample preparation and analysis have been improved through the use of auto-samplers, which have enhanced the accuracy, repeatability, and productivity of urine analysis [137]. Optimised sampling and analysis conditions for VOCs in urine headspace analysis should be investigated, as has been done for faecal samples [147]. This could significantly improve the clinical applicability of such methods, in order to gain wide-spread use. 

Despite the challenges, there are promising developments in the field of VOC analysis, which indicate that this method has great potential to aid in the diagnosis of bacterial infection in the future. The continuous drive for miniaturisation, portability, high-throughput and rapid analysis is likely to produce a wide range of eNose and IMS designs which will offer great potential for a large diversity of detection capabilities [148,149,150]. Moreover, as sensing technologies continue to advance, a greater number of VOCs are likely to be detectable at lower concentrations [151]. The progress that has been made in recent decades was illustrated in a meta-analysis, using machine learning on the VOCs emitted by human microbial pathogens, extracted from papers published between 1977 and 2016. The analysis showed that a set of 18 VOCs was sufficient to distinguish between 11 different infectious agents, with an accuracy of 77% [152]. This suggests that the fundamental potential of using VOCs for diagnosing bacterial infections has been successfully demonstrated. Further work is required to build an extensive library of reliable VOC profiles, across different analytical platforms, which correspond with UTI pathogens.

Some of the papers presented in this review cultured the sample/pathogens for a short period of time. The use of direct analysis is associated with decreased sensitivity and specificity, since urine varies drastically across individuals and even in the same person at different collection times (affected by digestion of food, level of hydration and lifestyle [153,154,155,156]). However, even if the specificity and sensitivity were to be decreased by 10–15%, these results would be comparable to the urine dipstick test results, while providing crucial insight for the patient, since a targeted course of antibiotics could be started immediately. 

Following GC-MS methods, eNose and IMS technologies were evaluated for the diagnosis of UTI. A common criticism of eNose and IMS technology is that they are unable to determine the identity and concentration of individual VOCs, except for combined-technology instruments [60]. It has therefore been argued that this “black box” approach relies too heavily on pattern-recognition algorithms [157]. Moreover, this method cannot yield a targeted VOC biomarker test for the diagnosis of a particular condition or disease [158]. In this case, it is true that the eNose approach cannot identify unique biomarkers for UTI. However, it should be noted that unique biomarkers do not necessarily exist for individual UTI pathogens and that even if they did, they would be included as a constituent of the overall pattern analysed by the algorithms. Furthermore, as indicated in this review, pattern recognition has already demonstrated potential in determining the status of a sample and recognised the relevant pathogen, without VOC identification. Another disadvantage associated with eNoses is that, compared to classic culture, there is a lack of antibiotic resistance data. In future, it may be possible to use eNoses on urine samples, with antibiotics added to them for a period of time, to identify if a strain is susceptible or resistant. This information could drastically reduce the test time, from the current standard of 24–72 h. If this is not feasible, local antibiotic resistance profiles and data from clinical laboratories could be used to make predictions and recommendations for appropriate treatments, once a pathogen has been identified in a urine sample. Moreover, the classification of growth phase is possible and this can be used to calibrate the antibiotic dosage for treatment [129]. 

For VOC detection of UTIs to become routine in clinical use, a number of hurdles need to be overcome. For example, more information regarding bacterial VOCs and data analysis models are required. Moreover, clinical staff need to be educated about VOC urine headspace analysis, taught how to interpret results and receive training on how to use the relevant equipment. An advantage of eNoses and IMS systems is that they are relatively user-friendly. While eNoses do not recognise specific VOCs, the sensors can be chosen to respond to specific chemical classes, which could increase the accuracy of detection. This emphasises that some prior information regarding the VOCs of the urinary pathogens is required, even for eNose analysis. One of the advantages of eNoses is also considered a drawback for expanding the use of the technology—the versatility of devices makes it challenging to establish a standard. Since research groups all over the world are developing different types of eNoses (MOS, QMB, or CP-based), it is unclear which one should be routinely used for UTI VOC detection. A standard, commercially available, and CE-marked device would increase the probability of wide-spread use by clinics and laboratories. Currently, the hardware used for detection, as well as the software required for data analysis, is advanced enough to identify infected urine samples, the pathogen and possibly its susceptibility to antibiotics. Since eNoses can be used in point-of-care applications (e.g, bedside testing), with automated analysis and delivering results minutes after sample collection, they have significant potential to serve as a clinical diagnostic tool for UTI. Furthermore, the information provided by eNoses is objective and less prone to contamination during collection or handling steps.

One of the key challenges associated with diagnosing UTI is that symptoms may overlap with other conditions, such as haematuria—one of the most common signs of bladder cancer. In addition, patients with asymptomatic bacteriuria may not benefit from treatment. However, distinguishing it from UTI is often subjective, as what is classified as asymptomatic could be problematic when patients with catheters or defects of the bladder (neurological or anatomical) are taken into consideration [25]. Catheters are a risk factor for UTI and catheter-associated urinary tract infection (CAUTI) is the most common nosocomial infection worldwide [159]. This highlights the need for an accurate and objective test. Further investigations are necessary to analyse whether VOC analysis could be used for this application.

There are also other potential applications. For example, during pregnancy, a patient with UTI can show no symptoms, i.e., the usual signs, such as pain and burning sensation, might be absent. Cases of UTIs during pregnancy, even when asymptomatic, have been linked with preterm delivery, intrauterine growth retardation, low birth weight, maternal hypertension, pre-eclampsia, and anaemia [160]. The application of eNoses would be optimal for the routine testing of urine, e.g., during antenatal appointments. This could provide an effective method of detecting asymptomatic UTI during pregnancy. 

Currently, the aim of deploying these technologies is not to replace the existing methods. Instead, they are intended to complement the diagnostic process, by aiding clinicians and medics to make better and faster decisions. The recent developments in technology, making the devices portable, accessible for point-of-care, automation of data analysis, and overall ease of use, facilitates VOC detection for routine clinical testing. Both metabolite detection and sensor-based techniques, together with pattern recognition, provide a powerful tool for diagnosis. Further studies are required to investigate the ability of eNoses to distinguish between resistant and susceptible bacterial strains. All medical devices must go through clinical trials. As such, eNoses need to be registered, verified, and validated before they can be accepted for routine clinical use.

## 8. Methods

An electronic search was performed using GoogleScholar and PubMed databases. The search terms volatile organic compounds (VOCs), urinary tract infection (UTI), microbial, pathogen, and bacteria were used in combination with the Boolean operators AND and OR. Additional searches were conducted including common bacterial pathogens (e.g., UroPathogenic *Escherichia coli* and *Klebsiella pneumoniae*) and analytical platforms (e.g., electronic nose (eNose) and gas chromatography mass spectrometry (GC-MS)). Articles were selected for full-text examination based on the relevance of the title and abstract. In the initial screening 65 articles were selected, further searches and references in the initial articles resulted in selecting another 79 articles for full-text examination. From the investigated articles, 45 were included in this review, and 27 of those are incorporated in Table 3. Compounds found in the literature (23 articles) are summarised in Table 2, divided by chemical classification (compounds were only included once in the most relevant category). Nine articles that analysed pathogens isolated from a different location (lungs/blood) or grown in blood culture were also included to illustrate that the same VOCs are bacteria-related rather than location/media dependent. Either of the following criteria had to be met in order to be included in the review: (1) VOC analysis was used to discriminate infected urine samples (clinical or simulated) or (2) VOCs were used to examine a pathogen that could cause UTIs. Multiple publications of the same data were excluded.

## Figures and Tables

**Table 1:** Characteristics of Volatile Organic Compound Detection Technologies.

Technology	eNoses		IMS	GC-MS
	**CP**	**MOS**		
Portability	Good	Good	Good	Poor
Cost	Low	Low	Low	High
Trained personnel	No	No	No	Yes
Sample throughput	High	High	Medium	Low
Speed	Real-time	Real-time	Real-time	Off-line
Metabolite detection	No	No	No ^1^	Yes
Pattern recognition	Yes	Yes	Yes	Yes
Chemical insight	No	No	Yes	Yes
Sensor drift	Yes	Yes	Minor	Minor

^1^ Some IMS constructs allow for metabolite detection. Abbreviations: Electronic noses—eNoses; Ion mobility spectrometry—IMS; Gas chromatography—ion mobility spectrometry—GC-IMS; Conducting polymer—CP; Metal oxide sensors—MOS.

**Table 2:** Volatile Compounds of Different Urinary Tract Infection Pathogens.

Compound	EC	PM	EF	PA	SA	KP	Compound	EC	PM	EF	PA	SA	KP
**Alcohol**							**Ester**						
Ethanol	√	√		√	√	√	ethyl acetate	√	√		√	√	√
Methanol	√						N-propylacetate	√		√			
Propanol	√						n-butyl acetate					√	
1-propanol	√						Ethyl phenylacetate	√					
N-propanol	√						isopentyl acetate		√		√	√	√
2-(Methylthio)-ethanol	√	√			√	√	3-Methylbutyl 2-methylpropanoate		√				
phenol	√				√		2-methylbutyl isobutyrate				√		
Butanol				√	√		2-Phenyl ethyl acetate		√				
1-butanol	√				√		Phenylethyl butyrate		√				
2-butanol		√		√	√	√	methyl 2-methylbutyrate				√		
Isobutanol		√		√	√	√	2-methylbutyl 2-methylbutyrate				√		
2-methyl-1-butanol	√						methyl methacrylate				√	√	
3-Methyl-1-butanol	√			√	√	√	ethyl 2-methylbutyrate				√		
octanol	√						isoamyl butyrate		√		√		
Decanol	√						amyl isovalerate				√		
1-Decanol	√						ethyl isovalerate					√	
dodecanol	√						ethyl formate					√	
Benzyl Alcohol	√						ethyl butanoate	√		√			
Isopentanol (isoamyl alcohol)		√		√	√	√	Propanoic acid 2-hydroxy-2-methyl-methyl ester					√	
phenethyl alcohol		√				√	ethyl formate					√	
ethylene glycol				√	√		Phenylacetic acid propylester	√					
4-methylphenol				√	√		**Ketone**						
2-methyl-1-propanol					√		acetone	√		√	√	√	
acetol (hydroxyacetone)					√		Acetoin (3-hydroxybutanone)	√				√	
2-phenylethyl alcohol					√		butanedione (diacetyl)	√					
methylbutenol				√	√	√	Butanone				√	√	√
							2-butanone	√		√	√		
**Aldehyde**							2,3-butanedione	√		√	√	√	√
Acetaldehyde	√				√		2-heptanone	√	√		√	√	√
Benzaldehyde	√				√		4-heptanone				√		
2-Heptenal	√				√		2-nonanone	√	√		√	√	√
(3-methylbutanal) Isovaleraldehyde	√	√		√	√	√	2-decanone	√					
2-methylbutanal	√		√		√		2-hexanone	√					
methylbutanal				√	√	√	2-Tridecanone	√					
isobutyraldehyde		√					2-undecanone	√	√		√	√	√
3-(ethylthio)-propanal				√			2,3-Heptanedione		√				
Propanal					√		2-pentanone			√	√	√	
3-methyl-2-butenal					√		2-aminoacetophenone	√			√		
2-ethylacrolein					√		3-octanone				√		
(Z)-2-methyl-2-butenal					√		methyl isobutylketone				√		
(E)-2-methyl-2-butenal					√		mercaptoacetone				√		
Methacrolein					√		1-hydroxy-2-propanone					√	
2-methylpropanal					√								
Nonanal					√		1-methyl-4-(1-methylethenyl)cyclohexane	√			√	√	√
formaldehyde	√		√	√	√	√							
							**Acids**						
**Hydrocarbons**							isobutyric acid					√	
Toluene	√	√	√		√	√	Butyric acid					√	
1-Methyl-naphthalene	√						4-methylhexanoic acid					√	
2-Methyl-naphthalene	√						Picolinic acid N-oxide	√					
Isoprene	√			√			Isoamyl benzoate		√				
2-Butene	√						**Sulphur**						
(Z)-2-butene					√		methyl mercaptan (Methanethiol)	√	√	√	√	√	√
(E)-2-butene					√		dimethyl disulfide	√	√	√	√	√	√
Undecane				√		√	dimethyl sulfide	√		√	√		√
2,4-dimethylheptane		√	√		√	√	dimethyl trisulfide	√	√		√	√	
3-methylheptane	√						Benzyl methyl sulfide		√				
undecene				√			2-methoxy-5-methylthiophene				√		
1-undecene				√			S-Methyl thiobenzoate		√				
2-methyl-2-butene				√			2,4-dithiapentane		√				
1,10-undecadiene				√									
1-nonene				√			**Chlorine**						
2-Nonene					√		Trichloromethane	√				√	
1-decene				√			4-Chloro-1H-indole	√					
1-dodecene				√									
Butane				√			**Inorganic**						
n-butane					√		ammonia	√		√		√	√
10-methyl-1-undecene				√			Hydrogen Sulfide	√		√			√
1,3-butadiene					√								
2-methylpropene					√		**Other**						
Propane					√		pyrazine derivative	√					√
2-Nonene, 3-methyl					√		benzene derivative	√	√		√	√	√
2,3,4-Trimethylhexane	√						phenol derivative	√	√				√
hexane	√						pentanone/toluene				√	√	√
2,3,3-trimethylpentane	√												
Acetic acid	√			√	√		**Nitrogen**						
Isovaleric acid					√		indole dimer	√					
2-methylbutyric acid					√		ethylamine		√				
							Indole	√			√		
**Nitrogen**							3-Methyl-1H-indole	√					
methylpyrazine	√			√			Cadaverine	√	√				
benzonitrile	√	√		√	√		putrescine		√				
2,3,5-trimethylpyrazine	√						Acetonitrile	√			√	√	
N-(Phenylmethylene)-methanamine	√						beta-phenylethylamine		√				
N,N′-Dibenzylideneethylenediamine	√												
N-(Phenylmethylene)-1-propanamine	√						**References**	[95]	[96]	[84]	[95]	[96]	[83]
N-(Phenylmethylene)-1-butanamine	√	√						[97]	[84]	[93]	[84]	[84]	[84]
n-nitrosodimethylamine		√						[83]	[83]		[93]	[93]	[96]
pyrrolidine		√						[80]	[98]		[99]	[99]	[93]
Isoamylamine		√						[81]	[81]		[100]	[100]	[100]
Isobutylamine		√						[93]	[101]		[102]	[103]	[104]
3-Methyl-N-(3-methylbutylidene)-1-butanamine		√						[99]	[77]		[105]	[106]	
Benzyl nitrile		√						[100]	[91]		[107]	[91]	
N-Butyl-benzenamine		√						[108]	[100]		[103]	[104]	
2-(3-Methylbutyl)-3,5-dimethylpyrazine		√		√				[105]			[106]		
3-Methyl-N-(2-phenylethylidene)-1-butanamine		√						[109]			[104]		
p-Pentylaniline		√						[110]					
N-(1,1-Dimethylethyl)-benzamide		√						[91,104]					
N-n-Butylphthalimide		√											
4-methyl-quinazoline				√									
Dimethylpyrazine				√									
pyrrole				√									
3-methylpyrrole				√									
1-vinyl aziridine				√									
Pyrimidine					√								
2-Acetylthiazole		√											
Trimethylamine	√	√				√							

Abbreviations: EC—Escherichia coli, PM—Proteus mirabilis, EF—Enterococcus faecalis, PA—Pseudomonas aeruginosa, SA—Staphylococcus aureus, KP—Klebsiella pneumoniae.

**Table 3:** Urinary Tract Infection and Pathogen Volatile Organic Compounds Detection.

Reference and Year	Technology	Number	Culture for Headspace Analysis	Experiment/Findings
Hayward et al. [77], 1977	Gas-liquid chromatograph	68 strains	Basal defined media with potassium lactate, amino acids and salts. Nutrient Broth (Oxoid) and MacConkey agar (Oxoid). Incubation still/shaken (160 rpm) at 37 °C	Samples were mechanically shaken for 5 min at 37 °C followed by 3 min incubation at 60 °C prior to analysis. It was found that *Proteus* species will produce dimethyl disulfide and methyl mercaptan from L-methionine.
Hayward et al. [78], 1977	Gas-liquid chromatograph	14 bacterial species	Basal defined media with lactate, amino acids, salts and vitamins cultured still at 37 °C or yeast extract broth still/shaken at 160 rpm.	*Proteus mirabilis* produces dimethyl disulfide and methyl mercaptan when cultured with L-methionine. *E. coli* produces ethanol from lactose. Detection of *E. coli* and *P. mirabilis* in urine based on ethanol production was achieved in 5 h (ethanol for *E. coli* methyl mercaptan for *P. mirabilis*) or 4 h (dimethyl disulfide for *P. mirabilis*). Other bacteria tested for dimethyl disulfide, methyl mercaptan or ethanol production were: *C. freundii*, *E. cloacae*, *S. marcescens*, *K. aerogenes*, *K, oxytoca*, *K. ozaenae*, *P. aeruginosa*, *S. aureus*, *S. epidermidis*, *S. marcescens* and *S. faecalis*. Samples were mechanically shaken for 5 min at 37 °C followed by 3 min incubation at 60 °C prior to analysis.
Coloe et al. [80], 1978	Gas-liquid chromatograph	49 strains	24 h, unshaken cultures. Media: arabinose, amino acid mixture, salt mixture, pH 7.4, nicotinic acid (0.5 mg) and calcium pantothenate	39/49 clinical samples: urine—29, faeces—8, pus—1, sputum—1. *E. coli* H19 incubation resulted in ethanol production from arabinose (maximum concentration after 8 h). For headspace analysis the mixture (culture fluid with 6 g of anhydrous K_2_CO_3_) was shaken at 160 rpm at 37 °C for 5 min and then heated at 60 °C in a water bath for 5 min.
Coloe et al. [81], 1978	Gas-liquid chromatograph	122 urine samples	37 °C, 4 h shaking in 6 mL yeast-extract peptone water enriched with arabinose and methionine	94 samples from UTI suspected patients, 28 uninfected controls. Results available in 4 h. *E. coli* produces ethanol from arabinose and *P. mirabilis* dimethyl disulfide from methionine.
Hayward et al. [128], 1983	Gas-liquid chromatograph	382 urine samples	Methionine yeast-extract peptone medium supplemented with arabinose incubated for 3.5 h.	*E. coli*, *Klebsiella*, *Citrobacter* and *Proteus* species. Ethanol production from lactose/arabinose of *E. coli* and methyl mercaptan from methionine of *Proteus* spp (methyl mercaptan oxidizes to dimethyl disulfide). No false positives and false negatives. Liquid was heated to 60 °C for headspace sampling.
Manja et al. [82], 1983	Gas-chromatography	96 urine samples	*E. coli* detection M-9 salt mixture, M-9L medium at 44 °C, 5 h. *Klebsiella* detection M-9A medium (M-9L with lactose replaced by adonitol and carbenicillin), 37 °C.	16 cases caused by *E coli*, 4 by *Klebsiella* and 2 were missed by GC. Ethanol presence indicates the bacteria present (in M-9L of *E. coli* and in M-9A of *Klebsiella*). All samples cultured in both media.
Davies et al. [83], 1984	Gas-liquid chromatograph	125 strains	Urine culture, 37 °C, 3.5 h, unshaken, media made of yeast-extract peptone medium concentrate.	For headspace samples—liquid temperature 60 °C for 5 min. Production of trimethylamine from acetylcholine biomarker for *P. mirabilis, P. vulgaris*, *P. retgeri*, *P. inconstants* A (not *in P. morganii* and *P. inconstans* B). Same species also produced ethanol and ethyl acetate. Ethanol production from arabinose was found in *E. coli*, *K. aerogenes*, all strains of *S. faecalis*. Ethyl acetate produced by *K. aerogenes*, *S. faecalis*, *E. coli* (with n-propanol) and *P. inconstans* B. No VOCs found *P. morganii*, *S. epidermidis*, *S. aureus*, *P. aeruginosa*.
Aathithan et al. [122], 2001	Osmetech Microbial Analyzer (OMA) Polymer sensor array	534 clinical samples	No	Sensitivity of 83.48%, specificity of 87.59% (infection defined as >1 × 10^5^ CFU/mL). 72.3% sensitivity and 89.38% Specificity at 10^4^ CFU/mL cut-off. Threshold PCA values were set using control experiments with reconstituted urine specimens inoculated with bacteria (4 cultures for each organism—result average 4 replicates)—4 blanks for each organism. Organisms: *E. coli*, *E. faecalis*, *S. aureus*, *Klebsiella* spp., *S. saprophyticus* and *P. mirabilis*
Pavlou et al. [123], 2002	Bloodhound BH114	25 and 45 urine samples	4.5 h incubation in enhanced media at 37 °C. Media included: 60% brain heart infusion broth, 40% cooked meat broth.	37 °C water bath for VOC sampling. First experiment: 20 out of 25 samples UTI confirmed—9 *E. coli*, 5 *P. mirabilis*, 6 mixed infection of gram positive cocci and *Proteus* species, 5 normal urine samples. Genetic Algorithms, Back-Propagation Neural Networks (GA-NN) used—100% prediction on the training set, the 9 samples not used for training were successfully identified. Second experiment: 30 out of 45 samples UTI—13 *E.coli*, 9 *Proteus*. spp, 8 coagulase negative *Staphylococcus* species. First NN 98% prediction rate, 13/14 unknown samples identified correctly. Second NN 95% prediction rate—18/19 unknown identified.
Yates et al. [125], 2005	Cyranose 320 electronic nose and Agilent 4440 Chemosensor	189 Sensor Responses for Urine (Cyranose 320)	No	Data reduction and optimisation using non-linear model with a kernel width parameter achieved 80% accuracy. Different statistical methods were used accuracy attained: ARX model 65%, 67% (using data from 19/32 sensors), 71% using the most negatively correlated sensor; RBF—50%, using correlation results 65%, Hybrid NARX model—80%. (73% when sensor number was reduced).
Yates et al. [129], 2005	Agilent 4440	28 and 40 samples for *S. aureus*. 32 data points for *E. coli*	Yes	Agilent 4440 was used along with a pattern recognition algorithm (radial basis function network) to distinguish between methicillin resistant and susceptible *S. aureus* (100% accuracy obtained) and identify the growth phase of *E. coli* (68.75% and 81.25% accuracy)—however the ones missed were misclassified by one growth stage.
Kodogiannis et al. [124], 2008	Bloodhound BH114	45 urine samples	Culture with Volatile generating kit, 5 h incubation at 37 °C	Headspace sample—37 °C water bath. 45 samples, 30 UTI (confirmed by microscopy + culture)—13 *E. coli*, 9 *Proteus* spp, 8 coagulase—*Staphylococcus* spp. 31 samples used for training, 14 for validation. Averaging the output of 4 feature-based networks (Divergence, Absorption, Desorption, Area) the overall accuracy was 100%.
Bruins et al. [130], 2009	MonoNose	52 strains	BD-BACTEC™–Plus-Anaerobic/FMedium with the addition of 0.1 mM FeCl_3_ for measurements. Other commercially available culture broths with different chemicals added were also tested	104 measurements taken. Bacterial species tested: *Escherichia coli, Proteus mirabilis, Enterococcus faecalis, Pseudomonas aeruginosa, Klebsiella pneumoniae, Staphylococcus aureus, Klebsiella oxytoca, Enterobacter cloacae, Clostridium difficile* and *Salmonella enteriditis, Salmonella typhimurium.* Specificity varied with the organism tested—between 67% and 100%.
Storer et al. [84], 2011	SIFT-MS	90 (10 replicate samples for each microbe)	Inoculation of sterile urine for 6 h.	10 samples of inoculated urine for each pathogen: *E. coli*, *Proteus vulgaris*, *P. aeruginosa*, *S. aureus*, *S. epidermidis*, *K. pneumoniae*, *E. faecalis*, *or Candida albicans*. Presents a table with mean concentration of different VOCs in the urine headspace.
Thorn et al. [85], 2011	SIFT-MS	11 strains (66—3 repeats for each strain at 5 h and 24 h)	Bacterial plate cultures (20–24 h after inoculation) were emulsified in 5 mL of 1% tryptone-0.5% yeast extract broth, incubated at 37 °C, orbital shaking, 200 rpm, 24 h.	*P. aeruginosa*, *S. aureus*, *E. coli*, *P. mirabilis*, *Burkholderia cepacian*, *S. pyogenes* and *E. faecalis* were studied. Control media sampling was also used (*n* = 36). Table with the different VOCs presented and discrimination between species is possible.
Wiesner et al. [94], 2011	IMR-MS	4 strains	Liquid cultures in Muller-Hinton medium shaken at 200 rpm and 37 °C	Antibiotic sensitive/resistant bacteria strain identification. Two strains of *E. Coli* (one sensitive and one ampicillin resistant) and two *S. aureus* strains (oxacillin sensitive/resistant) were used.
Bean et al. [86], 2012	GCxGC-TOF-MS	2 PA14 cultures and one LB-Lennox blank	24 h culture at 37 °C in lysogeny broth, Lennox	Discovered 28 new volatiles for *P. aeruginosa* PA14, 56 compounds in total. Samples heated and stirred for 10 min at 50 °C. Solid-phase microextraction SPME was used for headspace sampling PA14 strains.
Jünger et al. [100], 2012	MCC-IMS	15 human pathogens	24 h at 37 °C on Columbia sheep blood agar.	Detection of VOC for *E. coli*, *P. aeruginosa*, *P. mirabilis*, *K. pneumoniae*, *S. aureus*, S. *epidermidis* and other species using MCC-IMS in negative and positive ion mode as well as GC/MS for confirmation.
Sabeel et al. [126], 2013	Cyranose 320	13 urine samples, selected 9	No	Cyranose 320 was introduced in 2 mL of urine 10 times for each of the 13 samples. PCA was used for analysis (first component explained 97.087% of the variation). 9 samples were selected and classified as healthy (2), containing bacteria (1), containing mucus (9)—UTI marker.
Roine et al. [127], 2014	ChemPro 100i, Environics Inc., Mikkeli, Finland	101 Cultures from clinical samples	Cysteine lactose electrolytedeficient (CLED) medium	Pathogens: *E. Coli*, *S. saprophyticus*, *Klebsiella* spp, *E. faecalis*. Samples introduced in 36 °C water bath for 15 min prior to headspace analysis. LDA and LR were used. Infected/non-infected discrimination LR—95% sensitivity, 97% specificity; LDA—90% sensitivity, 96% specificity. Bacterial discrimination 95% sensitivity 96% specificity LDA. Validation method leave one out (LOOCV).
Boots et al. [104], 2014	GC-MS	200 (40 cultures for each bacteria)	Overnight culture, 37 °C, blood agar plates. Bacteria transferred to sterile brain heart infusion broth for 4 h, agitated at 37 °C.	4 bacteria studied: *P. aeruginosa*, *S. aureus*, *E. coli*, *K. pneumoniae*. After the overnight incubation the flasks were flushed with high-grade nitrogen (3000 mL over 15 min). Identification of the species is possible as well as methicillin-susceptible/resistant *S. aureus* strains.
Karami et al. [87], 2017	GC-MS	18 (2 medium used, 3 time points, 3 organisms)	24 h in nutrient agar, subcultured at 37 °C in two different broth medium—Muller Hinton Broth and tryptic soy broth.	Identifying VOCs of *E. coli*, *Candida albicans, S. aureus* from cultures. For headspace extraction cultures placed on a magnetic stirrer hotplate at 70 °C. Headspace extracted using SPME at 2, 4 and 24 h.
Ratiu et al. [91], 2017	ChemPro-100i (Environics Oy, Finland)and TD-GC-MS (for confirmation)	90 (3 replicates at 3 time points for each of the 10 cultures) and 30 for confirmation	Agar growth medium, 30 °C for *B. subtilis* and *S. aureus* and 37 °C for *E. coli*	540 headspace samples were analysed (270 from bacteria and 270 blanks with medium only). Discrimination between blanks and culture, the three species: *E. coli*, *Bacillus subtilis* and *S. aureus* and the different time points (24, 48 and 72 h) was possible. A list of VOC identified with TD-GC-MS is also shown.
DeJong et al. [92], 2017	Surface enhanced Raman spectroscopy	3 Strains	Liquid Culture in Tryptic Soy Broth Soybean-Casein Digest medium for 16 h at 37 °C transferred to agar-coated plates (Tryptic Soy Agar Soybean-casein Digest). SERS substrate was added.	The technique was applied to both cultures and simulated urine and blood infections with discrimination possible after 16 h.
Rees et al. [93], 2018	GCxGC-TOF-MS	100 clinical isolates (blood/urine)	37 °C 200 rpm shaking overnight in Difco Mueller-Hinston Broth (pre-culture) and 1:1000 same conditions for 12 h.	Prior to headspace analysis: 60 min at 37 °C, 200 rpm. Discrimination between the most common infectious pathogens in blood and urine (*E. coli*, *Klebsiella* species, *P. aeruginosa*, *P. mirabilis*, *S. aureus*, coagulase-negative *Staphylococcus*, *Acinetobacter* species, *Candida* species, *Enterobacter* spp, *Enterococcus* spp,.) was possible with a 95% accuracy using machine learning (random forest) on 203 VOCs or 96% accuracy with 811 VOCs.
Smart et al. [89], 2019	TD-GC-MS	86 chromatograms, 18 bacterial isolates	Overnight cultures, shaking at 180 rpm, 37 °C. Subcultures grown for 3 h. Antibiotics (cephalexin or ciprofloxacin) added after 2.5 h.	Bacteria: *E. coli*, *K. pneumoniae*, *P. aeruginosa*. Cultures grown in the presence of cephalexin or ciprofloxacin before headspace analysis. Difference in VOC profile was found between resistant and sensitive bacteria
Adebiyi et al. [90], 2019	IBM Electronic Volatile Analyzer	-	5-day culture in normal human urine at 37 °C	Concentration of bacteria 1 × 10^9^ CFU/mL. Incubated at 37 °C during sampling. Different models (LR, SVM, RF, MLP) had different accuracy (overall, 90%> and some had 100%)
